# GrpClassifierEC: a novel classification approach based on the ensemble clustering space

**DOI:** 10.1186/s13015-020-0162-7

**Published:** 2020-02-13

**Authors:** Loai Abdallah, Malik Yousef

**Affiliations:** 1 The Department of Information Systems, The Max Stern Yezreel Valley Academic College, Yezreel Valley, Israel; 2 The Department of Community Information Systems, Zefat Academic College, 13206 Zefat, Israel

**Keywords:** Ensemble clustering, Classification, k-means

## Abstract

**Background:**

Advances in molecular biology have resulted in big and complicated data sets, therefore a clustering approach that able to capture the actual structure and the hidden patterns of the data is required. Moreover, the geometric space may not reflects the actual similarity between the different objects. As a result, in this research we use clustering-based space that convert the geometric space of the molecular to a categorical space based on clustering results. Then we use this space for developing a new classification algorithm.

**Results:**

In this study, we propose a new classification method named *GrpClassifierEC* that replaces the given data space with categorical space based on ensemble clustering (EC). The EC space is defined by tracking the membership of the points over multiple runs of clustering algorithms. Different points that were included in the same clusters will be represented as a single point. Our algorithm classifies all these points as a single class. The similarity between two objects is defined as the number of times that these objects were not belong to the same cluster. In order to evaluate our suggested method, we compare its results to the *k* nearest neighbors, Decision tree and Random forest classification algorithms on several benchmark datasets. The results confirm that the suggested new algorithm *GrpClassifierEC* outperforms the other algorithms.

**Conclusions:**

Our algorithm can be integrated with many other algorithms. In this research, we use only the k-means clustering algorithm with different k values. In future research, we propose several directions: (1) checking the effect of the clustering algorithm to build an ensemble clustering space. (2) Finding poor clustering results based on the training data, (3) reducing the volume of the data by combining similar points based on the EC.

**Availability and implementation:**

The KNIME workflow, implementing *GrpClassifierEC*, is available at https://malikyousef.com

## Background

Clustering has a broad range of applications in life sciences and is used in many fields, from clinical information analysis to phylogeny and genomics and proteomics, over many years. The aim of clustering biological data is to cover the natural structure of the data and find important patterns within the data. Advances in molecular biology have resulted in big and complicated data sets, making clustering vital for information understanding and visualization. In addition, clustering can be a strong method to define the relationship between different samples (points) [1].

A clustering ensemble attempts to combine many clustering models to produce a better consistency and reliability result than that of individual clustering algorithms [2]. Cluster ensembles have been shown to be better than any standard clustering algorithm at improving accuracy and robustness across different data collections [3]. However, for clarification purposes in this study we are introducing the ensemble cluster (EC) algorithm that is different from the known clustering ensemble (CE).

The main assumption in this research is that points belonging to the same cluster are more similar to other points from other clusters even though their Euclidean distance is closer. This is because the clustering algorithms take into account both the geometric space as well as other statistical parameters.

In this research, the EC transformation algorithm is to run clustering algorithm (or multiple algorithms) several times with different parameter values where each run produce a categorical dimension (feature) of the new categorical data. For example running k-means with different value of *k, k* = 1,…,50, will generate a new categorical data with 50 dimensions.

Our current research presents a novel classification model that based on the Ensemble Cluster (EC) space. EC space is generated by EC transformation algorithm (See Algorithm 1 and Fig. 2) applied on a given data to generate a categorical data using clustering algorithms (one or more).

For example for a given point from the original data $X=({x}_{1},\dots , {x}_{n})$ with *n* features applying EC transformation using k-means over *k* = *1,…,50* will generate a new point $\widehat{X}=({c}_{1},\ldots , {c}_{50})$ in the new categorical space with 50 categorical values. The value of each ${c}_{i}$ indicates the cluster label that was assigned to the point in the $i\in \{1,\ldots,50\}$ iteration. Additionally, we can define an boolean identity function *id()* over the EC space between two categorical points $\widehat{X}=({c}_{1},\ldots , {c}_{50})$ and $\widehat{Y}=\left({v}_{1},\ldots , {v}_{50}\right)$

$$id\left({c}_{i},{v}_{i}\right)=\left\{\begin{array}{l}1 \quad if {c}_{i}={v}_{i} \\ \\ 0 \quad otherwise\end{array}\right.$$

$$Similarity\left(\widehat{X,} \widehat{Y}\right)=\frac{{\sum }_{i}^{n}id\left({c}_{i},{v}_{i}\right)}{n}$$

In other words, two points in the EC space are identical if they were assigned to the same clusters over all the iteration (k = 1,…,50). All the points that fall in the same cluster in the different clustering runs constitute a single group and are represented by a single point. Our algorithm classifies only the representors, and all the group members will have the same class label.

In general, one could use any clustering algorithm or a combination of algorithms. However, in our experiments, we use the *k-means* clustering algorithm with different *k* values. We have chosen the k-means as first step and as a future work; we would examine different algorithms and different combination to examine the impact on the performance of the algorithm. K-means is chosen for couple of reasons; firstly, it well known clustering algorithms, also we can specify the number of clusters, which is essential part to our algorithm and the differentiation between the different k values, is big. Interestingly, in our experiments, we observe that not only the number of the data points (size) decreased, but also the number of the generated features (categorical) is decreased. This reduction is different from traditional feature reduction that eliminates some of the unneeded features.

Combination clustering is a more challenging task than the combination of supervised classifications. Topchy et al. [4] and Strehl et al. [5] addressed this issue by formulating consensus functions that avoid an explicit solution to the correspondence problem. Recent studies have demonstrated that consensus clustering can be found using graph-based, statistical or information-theoretic methods without explicitly solving the label correspondence problem as mentioned in [6]. Other empirical consensus functions were also considered in [7–9].

A clustering-based learning method was proposed in [10]. In this study, several clustering algorithms are run to generate several (unsupervised) models. The learner then utilizes the labeled data to guess labels for entire clusters (assuming that all points in the same cluster have the same label). In this way, the algorithm forms a number of hypotheses. The one that minimizes the PAC-Bayesian boundary is chosen and used as the classifier. The authors assume that at least one of the clustering runs will produce a good classifier and that their algorithm will find it.

Clustering ensemble algorithms were also applied for semi-supervised classification [11, 12] based on the hypothesis that for noisy data they more accurately reflect the actual similarity between different objects. They propose a Co-association Matrix (CM) based on the outputs of different clustering algorithms and use this as a similarity matrix in the regularization framework. Berikon et al. [13] use the same idea in the semi-supervised regression method. They combine graph Laplacian regularization and cluster ensemble methodologies. To accelerate the calculation, they apply the low-rank decomposition of the CM.

Our method is different from those already published studies. We assume that the groups, which were built by the identical points in the categorical space, are relatively pure (i.e., all the points belonging to the same group have the same class).

Abdallah et al. [14, 15] developed a distance function based on ensemble clustering and use it within the framework of the k-nearest neighbor classifier and then improve selecting sampling for unsupervised data to be labeled by an expert. Additionally Abdallah and Yousef [16] integrated EC within Decision Trees, K Nearest Neighbors, and the Random Forest classifiers. The results obtained by applying EC on 10 datasets confirmed the hypothesis that embedding the EC space would improve the performance and reduce the feature space dramatically. However, in this research we do not integrated the EC with an existing algorithms, instead we suggest a novel classification method based on the categorical space that was received as a result of (EC).

A recent study by Yousef et al. [17] used EC classification comparing it to two-class SVM and one-class classifiers applied on sequence plant microRNA data. The results show that K-Nearest Neighbors-EC (KNN-ECC) outperforms all other methods. The results emphasize that the EC procedure contributes to building a stronger model for classification.

In this study we introduce a novel algorithm called *GrpClassifierEC* that based on EC transformation space. Several experiments were conducted in order to evaluate the performance of *GrpClassifierEC*. We tested it over 10 biological datasets and compare its results to the k-nearest neighbors, decision trees and random forest classification algorithms. The results show that the new algorithm *GrpClassifierEC* using the ensemble clustering was superior and outperforms the other baseline algorithms on most of the datasets.

## Methods

### The ensemble clustering transformation to categorical space

**Fig. 1:**
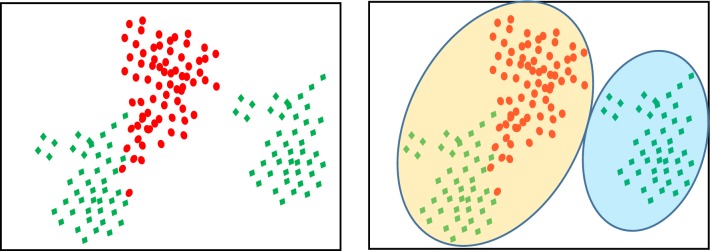
Example of clustering data

**Fig. 2:**
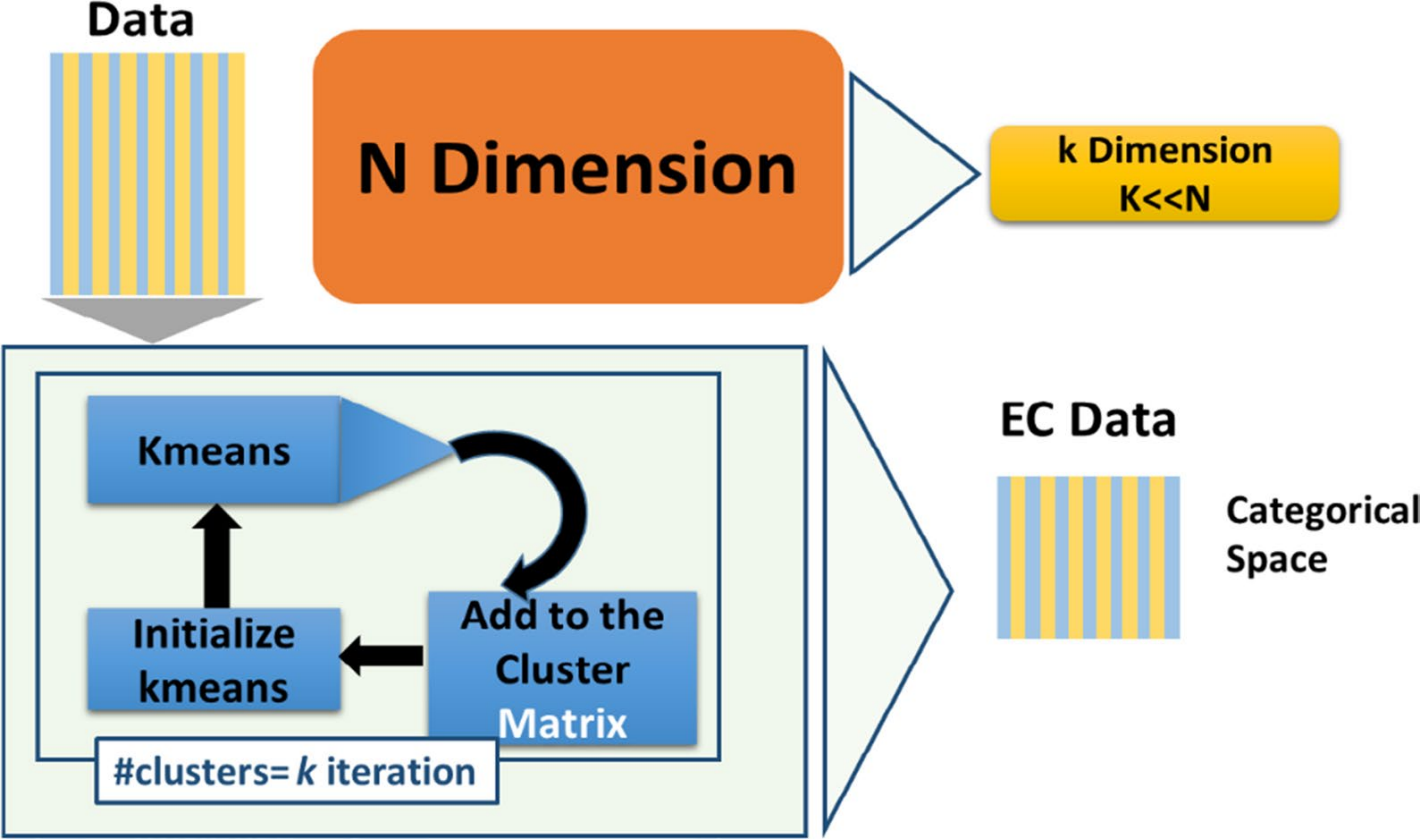
The workflow for creating the EC categorical space based on the k-means clustering algorithm. The original data is the input to the workflow. The outcome is a new dataset named EC data in a categorical space with dimension k. the sign ≪ indicates that *k* is dramatically smaller than the original data dimension N

This section describes the ensemble clustering (EC) transformation that transforms the original data from its original feature to categorical space as illustrated in Fig. 2. The basic algorithm assumes that points belonging to the same cluster are more similar than points that fall in different clusters. In real-world, this assumption may not always hold, as illustrated in the example presented in Fig. 1. In this example, the data includes two classes (circles and diamonds). If we cluster the data into two clusters, then the left cluster will include two types of classes and the right one will still have all the points from the same class.

As a conclusion, we decided to run the clustering algorithm several times. Points belonging to the same cluster in the multiple runs are consider as identical points and will define a $group$ that will be classified to the same class.

Let, $D$ be a set of labeled points used as training data, and *A* a set of unlabeled data. First, the *GrpClassifierEC* algorithm will create a new dataset $E$, where $E$ is a dataset combining $D$ and $A$ (i.e.,$E=D\cup A$), then the *GrpClassifierEC* runs the k-means clustering algorithm several times with different values of $k$ (we refer it to *nmc* = number of clusters) and creates the clustering matrix $cMat$. $cMat$ is a matrix where the ${i}^{th}$ row consists of the clustering results of the ${i}^{th}$ point in $E$. See Table 1 for an example of *cMat* with 20 points and 10 dimension of categorical features. The first column is the results of running k-means with k = 2 while the last column is the results of running k-means with k = 11. The values are the index of the cluster that was assigned by k-means. We record the results from *k* = 2.

**Table 1:** EC space for 20 points and number of cluster (nmc) of 11

Point/k	2	3	4	5	6	7	8	9	10	11
Point 1	c0	c2	c3	c2	c2	c4	c5	c4	c4	c5
Point 2	c0	c0	c3	c3	c2	c4	c4	c4	c4	c2
Point 3	c0	c2	c2	c4	c5	c5	c6	c6	c6	c6
Point 4	c1	c0	c0	c3	c3	c2	c2	c3	c3	c3
Point 5	c0	c0	c3	c3	c2	c2	c4	c2	c2	c2
Point 6	c0	c2	c3	c2	c4	c4	c5	c4	c4	c5
Point 7	c0	c2	c3	c2	c4	c4	c5	c5	c5	c4
Point 8	c0	c2	c2	c4	c4	c5	c6	c6	c6	c6
Point 9	c1	c0	c0	c3	c3	c2	c2	c3	c3	c3
Point 10	c0	c2	c3	c2	c4	c4	c5	c5	c4	c5
Point 11	c0	c2	c2	c2	c4	c5	c6	c5	c5	c4
Point 12	c0	c2	c2	c2	c4	c5	c6	c5	c5	c4
Point 13	c0	c2	c2	c2	c4	c5	c6	c5	c5	c4
Point 14	c0	c2	c3	c2	c2	c4	c5	c4	c4	c5
Point 15	c0	c2	c2	c2	c4	c5	c6	c5	c5	c4
Point 16	c0	c2	c3	c2	c4	c4	c5	c5	c4	c5
Point 17	c0	c2	c3	c2	c4	c5	c5	c5	c5	c4
Point 18	c0	c2	c3	c2	c2	c4	c5	c4	c4	c5
Point 19	c0	c0	c3	c3	c2	c2	c4	c2	c2	c2
Point 20	c0	c2	c2	c2	c4	c5	c6	c5	c5	c4

First column is the point name, second column is the results of assigning k-means of each point into two clusters (c0 and c1), the third column is the result of assigning k-means for each point into 3 clusters etc.

Applying the EC transformation on ${x}_{i}\in E$ will create a new point ${x}_{i}^{*}\in cMat$ with categorical values. The dimension of the *x*_i_^*^ is $k-1$*.* Therefore applying the EC transformation on the whole data will generate a new categorical data (EC data) that consists of *l* points with *nmc-1* categorical features.
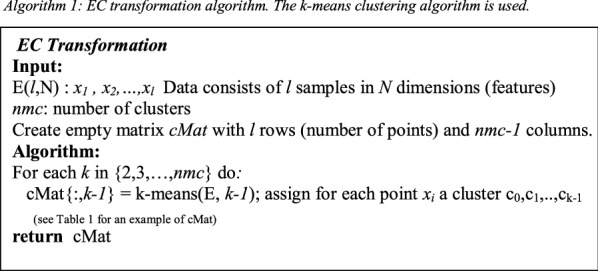


The new dimension *nmc-1*, usually, is much less that the original data dimension (*nmc-1* ≪  *N* in Fig. 2). More interestingly, the new EC data point can also be reduced as the new EC data contains identical points. We will explain it in more details in the section “Reduction of the Data”. Identical points that share the same clusters over the all iteration of *k*-means are represented as a same point in *cMat* as a result those points are *consider* to be one point, as a result all the identical points will define a group. For example, in Table 1, point 11, point 12 and point 20 have the same categorical values. This means, the vector space that represents those 3 points is = $g$(c0,c2,c2,c2,c4,c5,c6,c5,c5,c4). As a result, we consider those 3 points as a single point $g$ that we refer to it as a unique point. In other words, each group is represented by one unique point.

Note that, the set $E$ contains labeled and unlabeled points, and as a result, the groups may contain labeled and unlabeled points. Generally, there are three possible cases for the identical points in the same group:

1. The labeled points are having the same class label; the unlabeled points will be classified with this label.
2. The labeled points have different class labels: here the group points will be classified as the majority class.
3. All the points are not labeled: in this case, the group will be an unclassified group and the algorithm classifies it based on labeled nearest group.

To this end, we define a purity measurement for a given group in order to evaluate the purity of the grouping process. The purity measurement is based mainly on the probabilities of the labeled objects as follows:

$$purity\left({g}_{i}\right)=\mathop{{\sum }}\limits_{j=1}^{\#classes}{p}_{j}^{2}$$

where ${g}_{i}$ denotes group $i$ that was represented by vector ${g}_{i}$ in the matrix $G$, $\#classes$ denotes the number of the classes in ${g}_{i}$, and ${p}_{j}$ denotes the probability of class $j$ in group $i$. As can be seen, $purity({g}_{i})$ equals 1 when the group is pure and $\frac{1}{\#classes}$ for the lowest purity, that will decrease as the number of the classes increases.

The k-means algorithm is known to have a time complexity of *O(n *^*2*^*)* where *n* is the where *n* is the input data size. Then the complexity of the EC transformation is *O(k.n *^*2*^*)* where *k* is the number of times we run k-means. In fact, this part is the heaviest computation part of the *GrpClassifierEC* algorithm.

### ***GrpClassifierEC***—ensemble clustering based classifier

The *GrpClassifierEC* pseudo code is presented in *Algorithm 2*. The input to the classifier is the cMat matrix that generated by the EC transformation that described in Algorithm 1. The first step of the *GrpClassifierEC* is creating the *groups* extracted from *cMat*. *groups* = { $grou{p}_{i}$} where *i* = *1,…, s*. *s* is number of *groups*. The number of *groups* is influenced by *nmc*, the number of iteration that we run k-means. For instance, if we run k-means with *nmc* = *1* then all the points will be assigned to one cluster which means that we have just one group that contains all the data points. As we seen from Table 2 for the data Cercopithecidae vs Malvacea we have 449 groups with *nmc* = *30* while with the same data with *nmc* = *50* we have 593 groups (Table 3 #EC_Samples is equal to the number of groups). The number of groups is increasing as *nmc* is increasing and might reach the number of points in the data, which means that each group will host one point in categorical values.

**Table 2:** The data Cercopithecidae vs Malvacea with k = 30

Size	Unique points (groups)	#Points	Ratio unique points	Ratio all
1	305	305	67.929%	34.116%
2	68	136	30.290%	15.213%
3	22	66	14.699%	7.383%
4	18	72	16.036%	8.054%
5	11	55	12.249%	6.152%
6	5	30	6.682%	3.356%
7	5	35	7.795%	3.915%
10	4	40	8.909%	4.474%
13	3	39	8.686%	4.362%
8	3	24	5.345%	2.685%
9	2	18	4.009%	2.013%
29	1	29	6.459%	3.244%
14	1	14	3.118%	1.566%
31	1	31	6.904%	3.468%
Total	449	894		

The total number of points (points) is 894 which is the sum of column #Points. The size of the unique points is the sum of columns “Unique Points” which is 449. #Points is multiplication of Size and Unique Points. Ratio Unique Points is the #Unique Points/Total #Points while Ratio All is #Points/Total #Points

**Table 3:** GrpClassifierEC: -EC classifier results with a k value of 49 compared to Random forest applied on the EC samples and results for regular classifiers applied on the original data (K is number of clusters)

Data/performance	Data info			EC classifier GrpClassifierEC				Accuracy difference				EC-RF			Regular classifiers		
	#Point	#EC_Samples	Ratio	Sensitivity	Specificity	F-measure	Accuracy	EC random forest	Random forest	DTT	KNN	Sensitivity	Specificity	Accuracy	AccDT	AccKNN	AccRF
Aves vs embryophyta	1068	726	68%	0.97	0.92	0.97	0.96	0.02	0.01	0.05	0.02	0.84	0.97	0.93	0.91	0.93	0.95
Cercopithecidae vs Malvaceae	894	593	66%	0.98	0.97	0.98	0.98	0.08	0.05	0.10	0.07	0.84	0.94	0.90	0.88	0.91	0.93
Embryophyta vs Laurasiatheria	953	652	68%	0.96	0.92	0.96	0.95	0.08	0.04	0.10	0.07	0.94	0.72	0.87	0.85	0.88	0.91
Fabaceae vs Nematoda	2642	1004	38%	0.85	0.89	0.84	0.87	0.02	-0.01	0.04	0.00	0.92	0.76	0.85	0.83	0.88	0.89
Hexapoda vs Aves	2840	2087	73%	0.85	0.95	0.86	0.92	0.10	0.03	0.11	0.10	0.61	0.91	0.83	0.81	0.82	0.89
Laurasiatheria vs Brassicaceae	1209	570	47%	0.93	0.93	0.94	0.93	0.05	0.01	0.05	0.02	0.86	0.90	0.88	0.89	0.91	0.92
Malvaceae vs Fabaceae	1401	749	53%	0.69	0.87	0.68	0.82	0.16	0.05	0.15	0.12	0.84	0.22	0.67	0.67	0.70	0.77
brassicaceae vs Hexapoda	2584	870	34%	0.84	0.96	0.84	0.93	0.02	0.00	0.03	0.01	0.97	0.74	0.92	0.90	0.93	0.94
Hominidae vs Cercopithecidae	1829	1059	58%	0.72	0.91	0.73	0.86	0.15	0.09	0.20	0.14	0.25	0.87	0.70	0.66	0.71	0.76
Monocotyledons vs HomoSapiens	2625	1460	56%	0.92	0.93	0.92	0.92	0.10	0.03	0.09	0.04	0.84	0.82	0.83	0.83	0.88	0.89
Average			56%	87%	92%	87%	91%	8%	3%	9%	6%	79%	78%	84%	82%	85%	89%

Groups could have different sizes (size is the number of categorical points belongs to it). As seen from Table 2, group can have just one point; actually, we see that 305 different groups (unique points) with size 1 while 68 groups (unique points) with size 2. We see also that we have one group with size 31 which is the maximum size in this specific data.
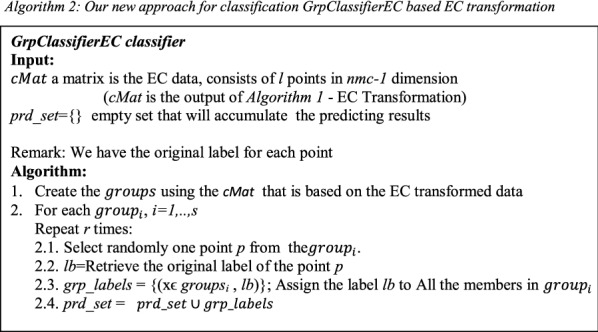


Following the step of creating the *groups*, we suggest our novel approach for classification, by randomly selecting *one* point from each group. The label of the selected point will be the label of all points belongs to the group. The process of selecting random point and assigning its label to its group repeated *r* times. The *GrpClassifierEC* classifier produce a list named *prd_set* that for contains the predictions results. Then in order to calculate the performances we run a scorer function. The scorer function compare the assigned label and original label for each point in order to get the confusion matrix. Accuracy statistics such as True-Positives, False-Positives, True-Negatives, False-Negatives, Recall, Precision, Sensitivity, Specificity, F-measure, as well as the overall accuracy and Cohen's kappa, are calculated.

### Reduction of the data

Table 2 shows the output of the EC procedure with *k* = 30 applied on the data Cercopithecidae vs Malvacea that contains 894 examples (points). The table also shows that the EC data has 449 unique points or groups, a 50% reduction in the size of the original data (449/894 = 0.5).

For each group (unique point), we measure its size, equal to the number of times this unique point appears in the EC data. For example, in Table 2, we have 305 unique points with size 1. All these points appear once in the new data space. In addition, we have 68 unique points. If each one appears twice in the data, then each one is size 2. There are 22 points with size 3—each of these 22 unique points appears 3 times in the data. Note that the labels are not included in the EC data. This means that the group of points at the EC space can have different labels associated with the original points and still share the same group.

**Fig. 3:**
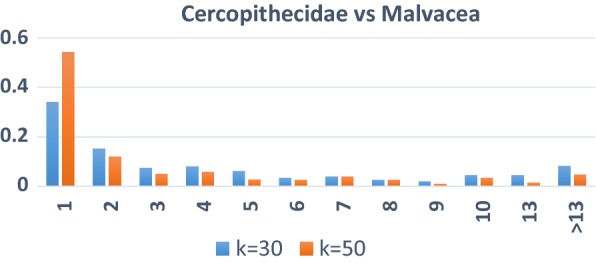
Distribution of the groups points (points) size comparing *nmc* = 30 and *nmc* = 50

Figure 3, shows the distribution of the group size for *nmc* = 30 and *nmc* = 50, and clearly indicates that as *nmc* increases, the number of groups with size 1 also increases. The expectation is that the number of groups of size of 1 should be the same as the number of the original number of points as we increase the value of *nmc*. In other words, each point will be hosted in one cluster. This actually raises a scientific question: what is the optimal value of *nmc* that will yield in improving the performance of the classifier, or more specifically, capture the nature of the data in terms of clusters. Answering this question is requiring additional future research.

#### Experiments on numerical datasets

To evaluate the performance of the new classifier *GrpClassifierEC* we compared its results to the k-nearest neighbors, decision trees and random forest classification algorithms. We tested it over 10 biological datasets and we compared the performance for each algorithm. The results show that the new algorithm using the ensemble clustering was superior and outperforms the other baseline algorithms on most the datasets.

### Datasets

The data consists of microRNA precursor sequences, and each sequence is made up of 4 nucleotide letters {A,U,C,G,}. The length of each precursor sequence is about 70 nucleotides. The source of this data is miRbase [18]. Part of the data we have used has was from other different studies [19–21], including our study [16].

One simple way of representing sequences that consist of 4 nucleotide letters is by employing the k-mers frequency. The $k$-mer counts in a given sequence were normalized by the length of the sequence.

Our features include k-mer frequencies, other distance features that were recently suggested by Yousef et al. [19] and secondary features suggested suggest by [22]. Many additional features describing pre-miRNAs have also been proposed [23] and are included in the feature set that numbers1038 features.

The main data consists of information from 15 clades (Table 4). The *Homo sapiens* sequences were taken out of the data of its clade Hominidae. The homology sequences were removed from the dataset and only one representative was kept. Each clade can serve as a positive examples or a as a negative examples. Considering all the different combination of pair of clades (positive/negative) it is possible to generate 256 datasets. We selected 10 datasets at random presented in Table 5.

**Table 4:** The table shows a list of clades used in the study

Data set	Number of precursors	Number of unique precursors
Hominidae	3629	1326
Brassicaceae	726	535
Hexapoda	3119	2050
Monocotyledons (Liliopsida)	1598	1402
Nematoda	1789	1632
Fabaceae	1313	1011
Pisces (Chondricthyes)	1530	682
Virus	306	295
Aves	948	790
Laurasiatheria	1205	675
Rodentia	1778	993
*Homo sapiens*	1828	1223
Cercopithecidae	631	503
Embryophyta	287	278
Malvaceae	458	419
Platyhelminthes	424	381

The first column represents the name of the clade, the second column the number of pre-cursors available on miRBase, and the third column the number of precursors after preprocessing the data

**Table 5:** Ten datasets

Positive data	Negative data
Aves	Embryophyta
Cercopithecidae	Malvaceae
Embryophyta	Laurasiatheria
Fabaceae	Nematoda
Hexapoda	Aves
Laurasiatheria	Brassicaceae
Malvaceae	Fabaceae
Brassicaceae	Hexapoda
Hominidae	Cercopithecidae
Monocotyledons	homoSapiens

The first column shows the name of the first clade positive data, and the second column the second clade negative data

### Implementation

We have implemented the GrpClassifierEC in Knime [24]. We have decided to use the free and open-source platform Knime due to its simplicity and very useful graphical presentations. Additionally, Knime is also a highly integrative tool. The Knime workflow consists from two parts, the first part is performing the EC transformation as describe on Algorithm 1. Actually, this part is time consuming where for example it took 13 min to generate the EC matrix for the input file that consists from 1038 features ad 1068 points. The run was performed on a laptop with Intell® Core ™ i7 7600U CPU @2.80 GHz 2.90 GHz with 16GM RAM.

### Model performance evaluation

We tested a different number of EC clusters using the k-means clustering algorithm with *nmc* values from 10 to 50. For each level, we performed 100 iterations with equal sample size, and then calculated the mean of each performance measurements described below.

For each established model we calculated a number of performance measures for the evaluation of the classifier such as sensitivity, specificity, and accuracy according to the following formulas (TP: True Positive, FP: False Positive, TN: True Negative, and FN False Negative classifications):

$$Sensitivity=\frac{TP}{TP+FN} (SE, \mathrm{r}\mathrm{e}\mathrm{c}\mathrm{a}\mathrm{l}\mathrm{l})$$

$$Specificity=\frac{TN}{TN+FP} (SP)$$

$$Sensitivity=\frac{TP+TN}{TP+FN+TN+FP} (ACC)$$

## Results and discussion

We also conducted a study comparing the new classifier *GrpClassifierEC* with the other known classifiers such as k-nearest neighbors, decision trees and random forest classifiers. The results are presented in Table 3. The results clearly show that the performance of the suggested classifier *GrpClassifierEC* was superior.

**Fig. 4:**
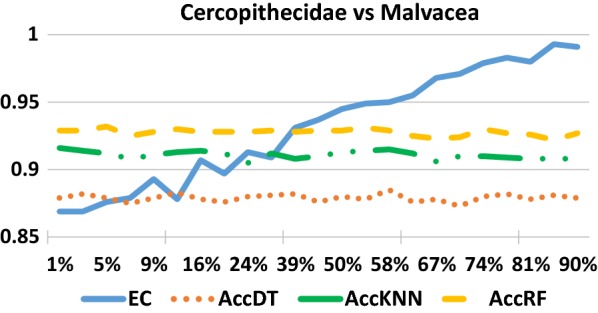
The accuracy of the classifiers over different level of sample training size

Figure 4 shows the performance of different classifiers at different levels of training percentage of the data. The results of EC refer to our own *GrpClassifierEC* classifier. We see that the performance is not significantly influenced by the size of the training part for the other classifiers while it does increase significantly for the GrpClassifierEC classifier, at the 39% level. In addition, performance can be improved significantly if the training part is increased, as a function of the value of k in the EC transformation.

In terms of data reduction, Tables 3 and 6 demonstrate that about 56% of the points data are reduced in the EC space with a *k* value of 49 and 39% in the EC space with a *k* value of 30. The results demonstrate the advantage of our approach in reducing the size of the data, for dealing with big data.

**Table 6:** GrpClassifierEC: EC classifier results with a k value of 30 compared to Random forest applied on the EC samples and results for regular classifiers applied on the original data

Data/performance	Data info			EC classifier GrpClassifierEC				Accuracy difference			
	**#Sample**	**#EC_Samples**	**ratio**	**Sensitivity**	**Specificity**	**F-measure**	**Accuracy**	**EC-RF**	**RF**	**DTT**	**KNN**
Aves vs Embryophyta	1068	513	48%	0.86	0.94	0.85	0.92	-0.01	-0.03	0.02	-0.01
Cercopithecidae vs Malvaceae	894	449	50%	0.94	0.92	0.94	0.94	0.04	0.01	0.06	0.03
Embryophyta vs Laurasiatheria	953	493	52%	0.94	0.83	0.94	0.91	0.04	0.00	0.06	0.03
Fabaceae vs Nematoda	2642	536	20%	0.78	0.88	0.79	0.84	-0.01	-0.05	0.01	-0.04
Hexapoda vs Aves	2840	1647	58%	0.76	0.92	0.78	0.88	0.05	-0.01	0.07	0.06
Laurasiatheria vs Brassicaceae	1209	406	34%	0.89	0.88	0.89	0.88	0.00	-0.04	0.00	-0.03
Malvaceae vs Fabaceae	1401	451	32%	0.55	0.80	0.53	0.73	0.07	-0.04	0.06	0.03
brassicaceae vs Hexapoda	2584	542	21%	0.77	0.95	0.78	0.91	-0.01	-0.03	0.01	-0.02
Hominidae vs Cercopithecidae	1829	786	43%	0.61	0.87	0.63	0.80	0.10	0.04	0.14	0.09
Monocotyledons vs HomoSapiens	2625	855	33%	0.86	0.87	0.86	0.87	0.04	-0.03	0.03	-0.01
Average			39%	80%	89%	80%	87%	3%	-2%	5%	1%

K is number of clusters. The section “Accuracy Difference” is EC Classifier-ACC of the other classifier. A positive value indicates that the EC classifier is better than the other corresponding classifiers. EC-RF is a random forest applied on the EC data, RF is a random forest applied on the original data. DTT is a decisionTrees while KNN is K- Nearest Neighbors applied on the original data

Tables 3 and 6 show the results of a comparison of the EC classifier with other classifiers applied on the whole feature space (named Regular Classifiers), and the performance of Random forest applied on the EC categorical data(EC-RF).

Table 3 presents results with a *k* value of 49, while Table 6 presents results with *k* 3. Interestingly, EC Classifier outperforms all the other approaches while using just 56% in average of the data (see ratio column), while the regular classifiers use 80% of the data for training. The EC classifier outperforms the standard approaches by 9% for the DT, 6% for the KNN, 8% for the random forest applied on the EC sample, and by 3% for the regular random forest.

The data in Table 6 show that one can reduce the size of the data to 39% ration with *k* = 30 and while still providing a reasonable result. The EC classifier outperforms DTT and EC-RF and KNN by 5%, 3% and 1% respectively, while RF outperforms it by 2%. More interestingly, that ratio of the reduction is an indication about the data redundancy and the similarity of the original data points.

## Conclusion

In this paper, we proposed a novel classifier based on ensemble clustering *GrpClassifierEC. Moreover,* we demonstrated the advantage of the EC approach in reducing the feature space and also in reducing the data size. Generally speaking, we shown that we are able to reduce the number of features dramatically to 5% or 3% (50/1038 = 0.048, 30/1038 = 0.028) and reduce the size of the data to 56% and 39%, and still achieve a similar performance level, or even outperform regular classifiers applied on the original data. However, to achieve these results the computation times that the EC transformation algorithm requires, increase.

The main assumption was that points within the same cluster share common traits more than points within different clusters. Thus, it may be more beneficial to represent objects based on the clustering space rather than the geometric space.

The approach suggested here is very useful for reducing the sample size and feature size when dealing with big data, while considering the EC data. For future research we will need to suggest an algorithm that would pick the optimal value of the clusters that and yield improved performance while reducing the size of the data considerably.

Our algorithm can be integrated with many other algorithms. In this research, we use only the k-means clustering algorithm with different k values. In future research, we propose several directions: (1) checking the effect of the clustering algorithm to build an ensemble clustering space. (2) Finding poor clustering results based on the training data, (3) reducing the volume of the data by combining similar points based on the EC. Additionally we will test it on gene expression data where the size of the features/genes is very large which might reach ten thousand of features.

## Data Availability

All of the sequence data was obtained from http://www.mirbase.org.

## Abbreviations

EC: Ensemble clustering
RF: Random forest
